# Medical Students' Competency and Confidence in Interpreting Electrocardiograms at King Faisal University, Al-Ahsa

**DOI:** 10.7759/cureus.46393

**Published:** 2023-10-03

**Authors:** Eman Elsheikh, Nurah Alkhteeb, Aisha Alamer, Maryam O Alarfaj, Ghaida AlQarni, Jumana Alsultan

**Affiliations:** 1 Internal Medicine, College of Medicine, King Faisal University, Al-Ahsa, SAU; 2 Cardiology, College of Medicine, Tanta University, Tanta, EGY; 3 Medicine, King Faisal University, Al-Ahsa, SAU; 4 Internal Medicine, King Faisal University, Al-Ahsa, SAU; 5 Medicine, King Faisal University, Al-Hofuf, SAU

**Keywords:** internal medicine, medical students, confidence, competency, ecg

## Abstract

Introduction

Electrocardiography is a crucial emergency tool in the pre-hospital situation. It is a useful non-invasive diagnostic technique for quickly identifying various heart disorders. The clinical value of the electrocardiogram (ECG) depends on the clinician's ability to interpret the result of the ECG accurately.

Aims

This study aims to assess the competency as well as the confidence in the interpretation of ECG among medical students at King Faisal University, Al Ahsa, Saudi Arabia.

Methods

This cross-sectional study was conducted among medical students enrolled at King Faisal University. Four hundred and ten (410) medical students from all five years completed an electronic self-administered pre-validated questionnaire. The questionnaire includes basic demographic data and ECG strips to assess medical students' competency and confidence levels in interpreting each case.

Results

More than half of the medical students were considered to have low competency (56.1%) and confidence (59%) levels. Increased competency and confidence scores were associated with fifth-year medical students and those who learned more about ECG interpretation through teaching during clinical rotations. The majority of medical students correctly interpreted anterior MI (69.3%), ventricular tachycardia (65.6%), and supraventricular tachycardia (61.2%). On the other hand, most students were unable to correctly identify pacemaker ECG (19.8%), long QT (21.2%) and left bundle branch block (33.4%).

Conclusion

Medical students' competency and confidence in ECG interpretation seems to be lacking. Fifth-year medical students who learned more ECG skills through teaching during clinical rotations tended to be more competent and confident with their ECG interpretation skills.

## Introduction

Electrocardiography is a crucial emergency tool in a pre-hospital situation [1]. It is a useful non-invasive diagnostic technique for quickly identifying a variety of heart disorders, particularly acute coronary syndrome and electrico-cardiac arrhythmias [2-3]. Patients with a risk of arrhythmias or suspected ischemic heart disease are frequently advised to have their ECGs monitored [4-5]. It can be handled properly to aid in the diagnosis or management of a number of life-threatening illnesses [1]. In critical care settings, including the emergency room (ER), intensive care unit (ICU), and cardiac care unit (CCU), patients heavily depend on health providers for treatment [6, 7]. Patients in these departments typically require ECG monitoring. Because of that, medical staff must gain the necessary knowledge and abilities to provide comprehensive and appropriate healthcare for all patients with various heart conditions, especially the critically ill in hospitals [8,9].

The clinical value of the ECG depends on the clinician's ability to accurately interpret the result of the electrocardiogram (ECG). Unreliable interpretation of the ECG may lead to poor management decisions which adversely affect patient outcomes [10]. Identification of potentially serious conditions, including acute myocardial infarction or other severe arrhythmias, is required immediately and accurately because these are time-dependent cases [11]. Therefore, health professionals and students who deal with patients must be capable of receiving and interpreting ECGs. If they are able to independently read a 12-lead ECG, they can anticipate any emergency treatment and offer the required interventions [12]. At King Faisal University, the academic department takes note of this significant issue and works to address it by implementing specific plans. First-year medical students start to learn early about the fundamentals of electrocardiogram (ECG) interpretation through introductory lectures. In the second and third year of the academic program, students learn more about ECG through a series of ECG workshops and lecturers, including both emergency and non-emergency scenarios. In the clinical years, students get the chance to enhance their electrocardiogram (ECG) interpretation skills during hospital rotations in Internal Medicine and Emergency Medicine by applying their skills on real patient cases. Different studies have shown that medical students and residents from numerous countries have difficulties interpreting ECGs [13]. However, there are few studies documenting the extent of this issue in universities of Saudi Arabia [13, 14]. In recent years, medical schools have shown a significant turning point in the development of undergraduate skills. Their primary concern was to identify the limitations of ECG interpretation and analyze the weak points among undergraduate students by implementing various strategies to improve ECG interpretation abilities throughout the medical education programs [14]. Thus, the aim of this study is to assess the competency and confidence in the interpretation of ECG among medical students at King Faisal University, Al Ahsa, Saudi Arabia.

## Materials and methods

This prospective cross-sectional study was conducted in the College of Medicine, King Faisal University, Saudi Arabia during the year 2023 using a self-administered, pre-validated questionnaire created by Vishnevsky et al. [10]. The ethical approval was granted by the Research Ethics Committee at King Faisal University (KFU-REC-2023-AUG-ETHICS1121). All male and female medical students in the five academic years (n=1383) were invited to complete the study questionnaire. A sample size of 301 was calculated using Raosoft Sample Size Calculator with a 95% confidence interval and a 5% margin of error. Participants were gathered in classroom halls and the electronic version of the questionnaire was distributed among the students using an online link. The responses (n=410) were voluntary and consent was taken from the students before starting the questionnaire. Students were supervised during the completion of the survey to ensure the authenticity in responses. The questionnaire is made up of two sections. The first section contains biographical data such as gender, year, GPA, and a question on their sources for learning ECGs. The second section contains eight ECG cases and students are asked to identify the diagnosis and the level of confidence in their answer. A final question asks about the overall confidence in interpreting ECGs.

Questionnaire criteria

The competency and confidence of medical students in interpreting ECGs have been assessed by using eight different ECG cases. The competency was evaluated by identifying the correct answer for each case. Each correct answer was coded with 1, while the incorrect answer was coded with 0. By summing up all eight items, we obtained the total competency score, with a possible score ranging from 0 to 8 points. The higher the score, the higher the competency in interpreting ECGs. By using 60% as a cutoff point to determine the level of competency, medical students were considered as having low competency if the score was 60% or below, and above 60% were considered as high competency levels. After evaluating competency levels, the assessment of confidence level was subsequently followed. This has been measured by 5-point Likert scale categories ranging from "not confident at all" coded with 1 to "extremely confident" coded with 5. The total confidence score was obtained by adding all eight items, generating a score ranging from 8 points to 40 points. The greater the score, the greater the confidence in interpreting ECGs. By following similar criteria as competency, medical students were categorized as having low confidence if the score was 60% or below, and above 60% were categorized as having high confidence levels.

Statistical analysis

Categorical data were presented using numbers and percentages (%). Continuous data were computed to present mean and standard deviation. The comparison of each correct ECG diagnosis in relation to the year of study has been conducted using the Chi-square test. The differences in the score of competency and confidence in relation to the basic demographic characteristics of the medical students have been performed using the Mann-Whitney Z-test and Kruskal-Wallis H test. Normality tests (i.e., statistical collinearity) were measured using Shapiro-Wilk test and Kolmogorov-Smirnov test. Based on the overall distribution of data, both the competency score and confidence score follow the non-normal distribution. Therefore, the non-parametric tests were applied. The Spearman correlation coefficient was also used to determine the correlation between the competency and confidence scores. A cutoff point of p<0.05 was used to indicate statistical significance. All data analyses were tabulated and calculated using SPSS version 26 (Statistical Packages for Social Sciences, IBM Corp., Armonk, NY).

## Results

This study enrolled 410 medical students. Table 1 describes medical students' basic demographic characteristics. Male students constitute 53.7%, with 5th-year students being the most common (40.5%). The most common source of ECG information was attendance in regular ECG lectures (88.3%).

**Table 1:** Basic demographic characteristics of medical students (n=410) † Variable with multiple response answers.

Study data	N (%)
Gender	
Male	220 (53.7%)
Female	190 (46.3%)
Academic year level	
First year	38 (09.3%)
Second year	59 (14.4%)
Third year	67 (16.3%)
Fourth year	80 (19.5%)
Fifth year	166 (40.5%)
Which of the following sources primarily contributed to your ECG interpretation skills ^†^	
Attendance in regular ECG lectures	362 (88.3%)
Teaching during Clinical Rotations	186 (45.4%)
Summer training clerkships	31 (07.6%)
ECG courses	66 (16.1%)
Self-study using printed materials (Textbooks)	98 (23.9%)
Self-study using web-based sources (online)	155 (37.8%)

**Figure 1:**
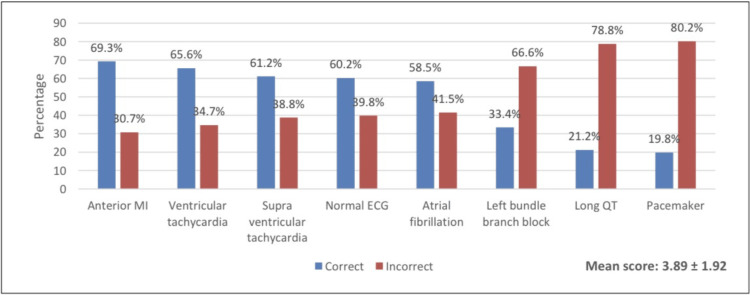
Results of ECG cases interpretation

In Figure 1, the highest rating for correct answers related to ECG case interpretation was anterior MI (69.3%), followed by ventricular tachycardia (65.6%) and supraventricular tachycardia (61.2%). The overall mean competency score based on eight ECG cases was 3.89 (SD 1.92). Based on the overall score, 56.1% were considered to have low competency (score ≤60%), and the rest (43.9%) had high competency levels (score >60%).

In Table 2, students were extremely confident about the diagnosis of ventricular tachycardia (35.4%), followed by anterior MI (27.3%), while they didn't have confidence in diagnosing Pacemaker (48.5%) and left bundle branch block (43.5%). The overall mean confidence score was 22.3 (SD 7.95). Low and high confidence levels were detected in 59% and 41%, respectively.

**Table 2:** Assessment of level of confidence toward ECG diagnosis (n=410)

Statement	N (%)
Degree of confidence in the diagnosis of anterior MI	
Not confident at all	82 (20.0%)
Slightly confident	61 (14.9%)
Somewhat confident	66 (16.1%)
Fairly confident	89 (21.7%)
Extremely confident	112 (27.3%)
Degree of confidence in the diagnosis of left bundle branch block	
Not confident at all	179 (43.7%)
Slightly confident	84 (20.5%)
Somewhat confident	82 (20.0%)
Fairly confident	32 (07.8%)
Extremely confident	33 (08.0%)
Degree of confidence in the diagnosis of ventricular tachycardia	
Not confident at all	64 (15.6%)
Slightly confident	64 (15.6%)
Somewhat confident	58 (14.1%)
Fairly confident	79 (19.3%)
Extremely confident	145 (35.4%)
Degree of confidence in the diagnosis of long QT	
Not confident at all	102 (24.9%)
Slightly confident	111 (27.1%)
Somewhat confident	111 (27.1%)
Fairly confident	48 (11.7%)
Extremely confident	38 (09.3%)
Degree of confidence in the diagnosis of normal ECG	
Not confident at all	90 (22.0%)
Slightly confident	97 (23.7%)
Somewhat confident	103 (25.1%)
Fairly confident	70 (17.1%)
Extremely confident	50 (12.2%)
Degree of confidence in the diagnosis of supraventricular tachycardia	
Not confident at all	62 (15.1%)
Slightly confident	81 (19.8%)
Somewhat confident	66 (16.1%)
Fairly confident	92 (22.4%)
Extremely confident	109 (26.6%)
Degree of confidence in the diagnosis of atrial fibrillation	
Not confident at all	106 (25.9%)
Slightly confident	74 (18.0%)
Somewhat confident	80 (19.5%)
Fairly confident	62 (15.1%)
Extremely confident	88 (21.5%)
Degree of confidence in the diagnosis of Pacemaker	
Not confident at all	199 (48.5%)
Slightly confident	72 (17.6%)
Somewhat confident	77 (18.7%)
Fairly confident	31 (07.6%)
Extremely confident	31 (07.6%)
Total confidence score (mean ± SD.)	22.3 ± 7.95
Level of confidence	
Low (score ≤60%)	242 (59.0%)
High (score >60%)	168 (41.0%)

**Figure 2:**
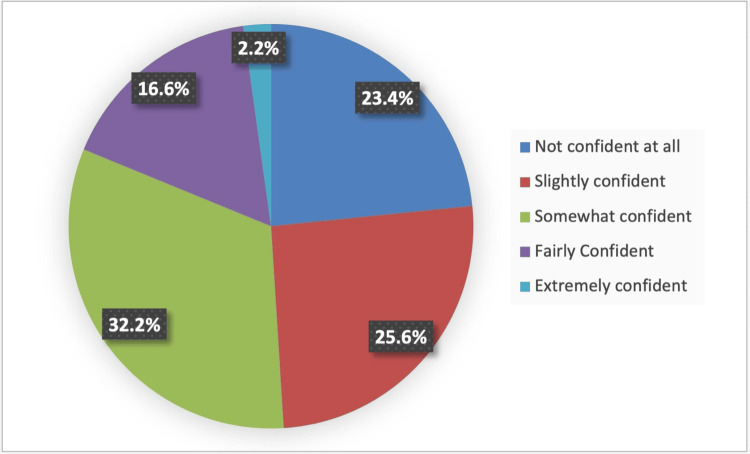
Overall confidence toward ECG interpretation skills

In Figure 2, the overall perceived confidence toward ECG interpretation skills was somewhat confident among 32.2%, slightly confident among 25.6%, 16.6% were fairly confident, 2.2% were extremely confident, and 23.4% were not confident at all.

**Figure 3:**
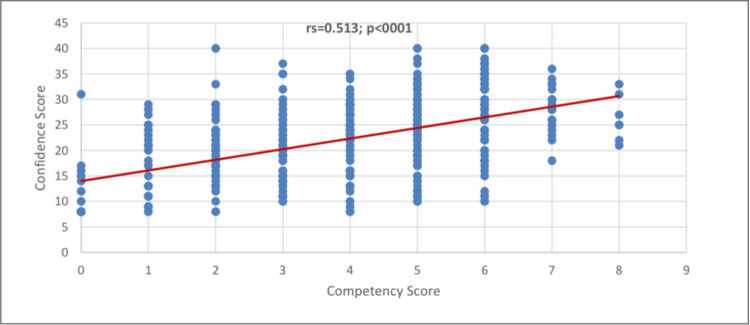
Correlation between competency score and confidence score

In Figure 3, the Spearman correlation coefficient indicates that there was a strong significant positive correlation between the confidence score and competency score (rs=0.513; p<0.001).

The study examined the relationship between demographic data of medical students and their scores in competency and confidence. As demonstrated in Table 3, a higher competency score was significantly associated with being a fifth-year student (H=122.5; p<0.001). Additionally, students who had access to ECG information through regular attendance in ECG lectures (Z=3.271; p=0.001) and teaching during clinical rotations (Z=6.227; p<0.001) demonstrated higher competency scores. Moreover, the findings indicate that a higher confidence score was significantly associated with male gender (Z=2.572; p=0.010), being a fifth-year student (H=109.4; p<0.001), and having sources of ECG information such as attending regular ECG lectures (Z=2.440; p=0.015), receiving teaching during clinical rotations (Z=7.094; p<0.001), engaging in self-study using printed materials (Z=2.355; p=0.019), and utilizing web-based sources for self-study (Z=2.018; p=0.044).

**Table 3:** Differences in the score of competency and confidence in relation to the Basic demographic characteristics of medical students (n=410) † Variable with multiple response answers. § P-value has been calculated using Mann-Whitney Z test. ‡ P-value has been calculated using Kruskal-Wallis H test. ** Significant at p<0.05 level.

Factor	Competency Score (8) Mean ± SD	Z-score	P-value^§^	Confidence Score (40) Mean ± SD	Z-score	P-value^§^
Gender						
Male	3.99 ± 1.95	0.833	0.405	23.3 ± 7.50	2.572	0.010^**^
Female	3.77 ± 1.89			21.2 ± 8.30		
Academic year level^‡^						
First year	3.95 ± 1.35	122.5		14.3 ± 5.04	109.4	
Second year	2.79 ± 1.89			20.5 ± 7.61		
Third year	1.93 ± 1.31		<0.001^**^	17.1 ± 6.49		<0.001^**^
Fourth year	4.51 ± 1.97			22.6 ± 7.22		
Fifth year	4.77 ± 1.42			26.7 ± 6.46		
Which of the following sources primarily contributed to your ECG interpretation skills^†^						
Attendance in regular ECG lectures	4.01 ± 1.89	3.271	0.001^**^	22.6 ± 7.91	2.440	0.015 ^**^
Teaching during Clinical Rotations	4.54 ± 1.80	6.227	<0.001^**^	25.3 ± 6.48	7.094	<0.001^**^
Summer training clerkships	3.94 ± 2.13	0.179	0.858	24.4 ± 7.51	1.458	0.145
ECG courses	3.56 ± 1.82	1.656	0.098	23.9 ± 7.74	1.636	0.102
Self-study using printed materials	4.03 ± 1.81	0.824	0.410	23.9 ± 7.39	2.355	0.019**
Self-study using web-based sources	3.75 ± 1.97	1.069	0.285	23.2 ± 7.64	2.018	0.044**

Table 4 presents the findings regarding the association between medical students' academic year and their ability to correctly diagnose various cardiac conditions. Specifically, fifth-year medical students demonstrated a significantly higher likelihood of providing accurate diagnoses for anterior myocardial infarction (p<0.001) and left bundle branch block (p<0.001). Conversely, first-year medical students exhibited a greater propensity for correctly diagnosing long QT (p<0.001), normal electrocardiogram (p=0.003), supra ventricular tachycardia (p<0.001), and atrial fibrillation (p<0.001). In addition, it was observed that fourth-year medical students had a significantly higher rate of correct diagnoses for ventricular tachycardia (p<0.001) and Pacemaker (p=0.001).

**Table 4:** Correct answers for each ECG diagnosis in relation to year of study (n=410) § P-value has been calculated using Chi-square test. ** Significant at p<0.05 level.

Diagnosis	Year of Study					P-value^§^
	1^st^ year N (%) (n=38)	2^nd^ year N (%) (n=59)	3^rd^ year N (%) (n=67)	4^th^ year N (%) (n=80)	5^th^ year N (%) (n=166)	
Anterior MI	15 (39.5%)	33 (55.9%)	21 (31.3%)	69 (86.3%)	146 (88.0%)	<0.001^**^
Left bundle branch block	03 (07.9%)	06 (10.2%)	12 (17.9%)	28 (35.0%)	88 (53.0%)	<0.001^**^
Ventricular tachycardia	16 (42.1%)	29 (49.2%)	15 (22.4%)	68 (85.0%)	141 (84.9%)	<0.001^**^
Long QT	20 (52.6%)	08 (13.6%)	11 (16.4%)	21 (26.3%)	27 (16.3%)	<0.001^**^
Normal ECG	32 (84.2%)	36 (61.0%)	30 (44.8%)	51 (63.7%)	98 (59.0%)	0.003^**^
Supra ventricular tachycardia	31 (81.6%)	23 (39.0%)	16 (23.9%)	54 (67.5%)	127 (76.5%)	<0.001^**^
Atrial Fibrillation	30 (78.9%)	24 (40.7%)	18 (26.9%)	47 (58.8%)	121 (72.9%)	<0.001^**^
Pacemaker	03 (07.9%)	06 (10.2%)	06 (09.0%)	23 (28.7%)	43 (25.9%)	0.001^**^

## Discussion

The undergraduate curriculum is a foundation of skills and development. Medical students at King Faisal University are initially introduced to the fundamentals of electrocardiogram (ECG) interpretation during their first year of medical education through didactic lectures. During the second and third years of their academic program, students undergo training in the interpretation of electrocardiograms (ECGs) through a series of ECG workshops and lectures. These educational sessions encompass a range of emergency and non-emergency scenarios, providing students with the necessary knowledge and skills to analyze and interpret ECGs effectively. The Year 4 and 5 clerkships in Internal Medicine and Emergency Medicine provide students with the opportunity to practice their abilities in electrocardiogram (ECG) interpretation within the context of hospital rotations, utilizing authentic patient cases and hospital ECG strips. The educational experience of senior medical students is also enhanced with the inclusion of lectures, seminars, and group discussions conducted within the college classrooms.

The present study investigated the competency and confidence levels of medical students in interpreting ECG strips. This study finds that medical students' competency in ECG interpretation was inadequate. Based on eight ECG cases, the overall mean competency score was 3.89 (SD 1.92) out of 8 points, with 56.1% considered as low levels, while 43.9% had high competency levels. These findings almost mirrored the study of Vishnevsky et al. [10], where the overall competency mean score was 3.23 (SD 1.81) out of 8 points, suggesting a low level of competency. This corroborates the study of Amini et al. [12], with a mean competency score of 5.13 (SD 2.25) out of 10 points. Surprisingly, a study conducted by Oluga [15] showed the least prevalence of low ECG skills competency, as only 2.8% of the medical students were able to interpret correctly at least 50% or more of the 22 ECG cases, adding that the medical students in University Nairobi have suboptimal competency levels in ECG interpretation skills. On the contrary, paramedic students seem to have better ECG competency skills. According to the report of Mobrad [16], 64.2% of the paramedic students score >7.5 points indicating competency in ECG interpretation. Consistent with this report, a study by Al Harbi et al. [17], documented adequate competency levels among emergency medicine residents reporting average levels at 63.5% and high levels of 24%, with only 12.5% considered low competency levels. There is a need to increase the ECG skill competency of medical students. ECG lectures and training sessions should be incorporated into the undergraduate curriculum to improve the level of competency of medical students in ECG interpretation skills.

Being a fifth-year level, attendance in regular ECG lectures and participation in teaching sessions during clinical rotation were the factors that increased competency levels. Consistent with our findings, a study conducted among medical students and interns in Taif [14] found that interest in ECG interpretation and previous attendance in ECG courses were significantly related to better performance; however, academic year and gender showed no significant association with ECG interpretation. According to multivariate analysis in Poland [13], being in clinical years and self-learning determined competency in ECG interpretation. Also, ECG self-learning was associated with higher competency in ECG interpretation, but they found no significant difference between students who attended or did not attend regular ECG classes (p=0.99). In a regional study conducted in multiple Arab countries [18], they documented that postgraduate residents in the third and second years have an increased likelihood of better knowledge of ECG interpretation than participants in the first year of residency. Thus, these accounts indicate that increasing competency levels could be associated with increasing academic year level and attendance to ECG lectures and teaching.

The competency levels mirrored the results of confidence. Approximately 59% of the medical students were regarded as having low confidence levels, with a total mean confidence score of 22.3 (SD 7.95) out of 40 points. In a study carried out among postgraduate foundation year 1 and 3rd-year medical students [19], they reported that respondents in the focused teaching program (FTP) group exhibited a greater improvement in confidence than the respondents of self-directed learning (SDL). However, among the medical interns in Al Ahsa [20], participants demonstrated insufficient performance in ECG interpretation despite completing advanced cardiac life support courses. Their performance as a whole did not significantly improve, believing that their institution did not do enough to train them in reading ECGs.

Gender male, fifth-year student, and having sources of ECG interpretation skills such as attendance in regular ECG lectures, teaching during clinical rotations, and self-study using printed materials and web-based sources were the significant factors for increased confidence. In a study conducted by Alghamdi et al. [14], ECG reading interest and ECG course participants were significantly associated with better performance in ECG interpretation; however, academic year and gender have no significant influence on ECG interpretation. Furthermore, our study noted a positive, strong, and significant correlation between the competency score and confidence score (p<0.001), suggesting the increased competency score will likely increase the confidence score. This mirrored the study of Getachew et al. [21], as they found that confidence levels were positively associated with competency levels.

Moreover, regarding the details of competency in interpreting each ECG strip, many of our subjects were able to correctly interpret ECG strips about anterior MI (69.3%), ventricular tachycardia (65.6%) and normal ECG (60.2%), which is similar to the results of Vishnevsky et al. [10] where many of their students were able to interpret anterior MI (81%), ventricular tachycardia (54%), and normal ECG (65%). On the other hand, in Iran [12], only a minority of students were able to identify normal sinus rhythm (36.2%) and acute myocardial infarction (22.3%).

In our study, poor ECG readings were seen in the left bundle branch block (33.4%), long QT (21.2%) and Pacemaker (19.8%). Poor performance in interpretation of left bundle branch block (19.8%) and long QT (14.1%) was also observed among newly graduated medical students in Kenya [15]. Identification of ECG diagnoses that are poorly interpreted by medical students, can help to emphasize and strengthen the teaching and training of these ECGs.

There is some variation in the types of ECGs answered correctly among different academic levels. Fifth-year medical students were seen to have better reading skills for anterior MI and left bundle branch block, and fourth-year students had better diagnosis skills in ventricular tachycardia and Pacemaker. Surprisingly, first-year medical students seem to understand better the diagnosis of normal ECG and atrial fibrillation than other medical students. In the first year of medical school, students are taught the fundamentals of interpreting an ECG and being able to identify the basic parts of a normal ECG is the core of their curriculum. This can explain the reason why most first-year medical students were able to identify a normal ECG (84.2%).

Our study is limited by the fact that it was conducted in a single academic institution where the curriculum is standardized for all students, which could make it difficult to assess the performance of Saudi students as a whole. In addition, the selection of ECGs is restricted to eight emergency and non-emergency cases, and additional ECG strips could be evaluated. Also, the fewer medical students in the pre-clinical years than those in the clinical years may influence certain results.

## Conclusions

There was a lack of competency and confidence in interpreting ECG among medical students. However, increased competency and confidence were more prevalent among fifth-year students who learned more ECG interpretation skills through teaching during clinical rotations. A case-based program can improve trainee confidence and competence toward ECG interpretation. ECG skills interpretation can be developed at any training level, particularly if guided by a dedicated teaching training program.
